# Vitreous Substitutes in Vitreoretinal Surgery: From Native Vitreous Physiology to Bioengineered Experimental Replacements

**DOI:** 10.3390/jfb17060301

**Published:** 2026-06-17

**Authors:** Alessandro Avitabile, Ludovica Cannizzaro, Dario Rusciano

**Affiliations:** 1 Neurovisual Science Technology (NEST), 95124 Catania, Italy; alessandro.avitabile@unipa.it; 2 Department of Biomedicine, Neuroscience and Advance Diagnostic (BiND), University of Palermo, 90128 Palermo, Italy; ludocann@gmail.com

**Keywords:** vitreous substitute, hydrogel vitreous implant, vitrectomy tamponade materials, biocompatible vitreous replacement, polymer-based vitreous body regeneration

## Abstract

The vitreous body is not only a transparent filling material of the posterior segment; it is a soft, hydrated, and biologically active matrix that supports structural, optical, and biochemical homeostasis. Vitrectomy therefore leaves a functional deficit that current substitutes only partly address. Intraocular gases, silicone oils, and perfluorocarbon liquids remain essential surgical tools, but they mainly provide mechanical tamponade and do not reproduce native viscoelasticity, diffusion control, or protection against oxidative and inflammatory stress. This review considers vitreous replacement as a functional biomaterials challenge. We discuss native vitreous physiology, the limitations of present tamponade agents, and emerging bioengineered substitutes designed to create a more physiological intravitreal environment. Particular attention is given to hydrogel and polymer-based systems, especially hyaluronic acid-based and in situ crosslinked platforms, which are being developed to combine optical clarity, injectability, soft mechanical support, controlled degradation, and favorable tissue interaction. We also emphasize the need for standardized preclinical testing of swelling, enzymatic stability, drug diffusion, rheology, and long-term biocompatibility. Although next-generation materials may move the field beyond passive space filling, manufacturing reproducibility, regulatory validation, chronic safety, and cautious early-phase trials remain major translational barriers.

## 1. Introduction

### 1.1. The Main Challenges of Vitreous Replacement

The vitreous body fills almost four-fifths of the human eye. In clinical practice, it is often described as the transparent gel of the posterior segment. This description is correct, but it is also too limited. The vitreous is not simply an inert filler. It is a highly hydrated and carefully organized extracellular matrix, with structural, optical, mechanical, and biochemical roles that are closely linked to retinal homeostasis [1,2]. Its framework consists mainly of water, collagens, hyaluronic acid, proteoglycans, soluble proteins, and resident hyalocytes. Together, these components form a delicate viscoelastic scaffold with mechanical and diffusional properties that are difficult to reproduce with artificial materials [3].

Several aspects of this organization are clinically relevant. Mechanically, the vitreous cushions eye movements, distributes forces within the globe, and helps maintain a stable relationship between the neurosensory retina and the retinal pigment epithelium. Optically, its high water content, sparse fibrillar architecture, and refractive compatibility with neighboring ocular media allow light to pass with minimal scattering. Biochemically, it influences the movement of oxygen, metabolites, cytokines, and other soluble mediators between the anterior and posterior segments, helping to preserve retinal homeostasis and limit oxidative imbalance [4,5]. When this structure changes with age, disease, or surgery, the consequences may include vitreous liquefaction, posterior vitreous detachment, vitreoretinal traction, retinal detachment, and interface disorders [5,6,7,8].

Pars plana vitrectomy has transformed the management of many sight-threatening diseases, including rhegmatogenous and tractional retinal detachment, macular holes, diabetic vitreous hemorrhage, and complex vitreoretinal interface disorders [9,10]. Yet this success has a biological cost that is sometimes underestimated. Removing the native vitreous also removes a tissue that normally contributes to ocular biomechanics, diffusion-mediated exchange, oxygen-gradient regulation, and oxidative buffering. The postoperative eye is therefore exposed to altered fluid movement, increased shear stress, disturbed metabolic exchange, and a higher oxidative burden. These changes may contribute to cataract progression, retinal toxicity, chronic inflammation, and proliferative vitreoretinopathy [4,5,11].

This is why the search for a true vitreous substitute remains important. A satisfactory replacement should do more than fill the vitreous cavity or temporarily hold the retina in place. Ideally, it should be optically clear, close to the native refractive index, mechanically soft and viscoelastic, permissive to controlled diffusion of oxygen and metabolites, and biologically tolerated over time [8,12]. It should also avoid toxic, inflammatory, or mechanically harmful interactions with the retina, lens, ciliary body, and trabecular outflow pathway. Current substitutes, including gases, silicone oils, and perfluorocarbon liquids, remain indispensable in vitreoretinal surgery. Still, they are essentially surgical tools. They can support retinal reattachment, but they do not restore the biochemical, diffusional, and protective functions of the native vitreous, and each carries its own material-specific limitations [5,12].

For these reasons, we believe that vitreous substitution should be considered primarily as a biomaterials problem, not only as a surgical one. The central question is not simply how to occupy the empty vitreous cavity after surgery, but how far it is possible to rebuild a more physiological intravitreal environment. Progress in polymer chemistry, hydrogel design, nanocomposites, and controlled drug delivery has made this question more realistic than it was in the past [8,13,14,15]. Bioengineered hydrogels and multifunctional polymer systems are now leading candidates because they can be tailored to approximate the hydration, softness, viscoelasticity, transparency, and diffusion properties of the natural vitreous.

Specifically, this review covers the physiological functions that are lost after vitrectomy, the material-specific strengths and limitations of currently used gases, silicone oils, heavy oils, semi-fluorinated systems, perfluorocarbon liquids, and balanced salt solutions, and the emerging hydrogel, polymer, hybrid, and bioactive platforms that are being developed as more physiological vitreous replacements.

### 1.2. Conceptual Framework

The present view of vitreous substitution has developed from three overlapping areas of evidence: studies of native vitreous physiology, clinical experience with existing endotamponades, and experimental work in biomaterials. Physiological studies showed early on that the intact vitreous helps regulate intraocular oxygen distribution, suggesting that a replacement should aim to restore function rather than provide only mechanical support [4]. Later reviews and translational studies refined the desirable properties of vitreous substitutes, placing emphasis on optical transparency, refractive compatibility, suitable viscoelasticity, long-term biocompatibility, and retinal safety [8].

Clinical experience has also been instructive. Gases, silicone oils, heavy tamponades, and perfluorocarbon liquids can be very effective in selected surgical situations, but their usefulness depends mainly on surface tension, buoyancy, density, or short-term intraoperative handling. Their limitations are equally familiar: postoperative positioning, emulsification, altered oxygen diffusion, intraocular-pressure changes, inflammation, retinal toxicity, and the need for removal in some cases [12,16]. This practical experience draws a clear line between a temporary endotamponade and a true vitreous substitute.

At the same time, preclinical studies and recent reviews of polymeric and hydrogel-based materials have shown that injectable and in situ-forming systems can be designed to provide optical clarity, tunable mechanics, prolonged residence, and improved compatibility with the intraocular environment [8,12,13]. These developments support a shift away from passive space filling and toward bioengineered substitutes intended to restore volume, retinal support, and at least part of the homeostatic function lost after vitrectomy. This review follows that line of reasoning. It examines the physiological rationale for vitreous replacement, the limits of current clinical substitutes, and the strategies through which next-generation biomaterials may reshape postoperative homeostasis in vitreoretinal surgery.

### 1.3. Literature Search and Article Selection

For this narrative review, we performed targeted searches of PubMed/MEDLINE, Scopus, and Web of Science, covering publications from database inception to April 2026. The search was intended to identify experimental, translational, and clinically relevant studies dealing with vitreous physiology, vitreoretinal surgery, and the development of vitreous substitutes.

The search terms covered native vitreous structure and function, including vitreous body, vitreoretinal interface, oxidative stress, and proliferative vitreoretinopathy. We also searched for surgical contexts such as pars plana vitrectomy, retinal detachment, macular hole, and vitreous tamponade; established clinical substitutes such as silicone oil, intraocular gases, and perfluorocarbon liquids; and biomaterial-based approaches such as vitreous substitutes, hydrogels, injectable polymers, thermogelling systems, and drug-delivery platforms. Additional terms, including biocompatibility, rheology, optical coherence tomography, and regulatory considerations, were used to capture studies relevant to translation.

Titles and abstracts were screened for relevance to material design, biological performance, or translational potential. We prioritized original experimental studies, mechanistically informative reviews, and translational reports that clarified either the structure-function relationship of the native vitreous or the performance of next-generation substitutes in preclinical models. Particular attention was given to studies published between 2024 and early 2026, especially those describing multifunctional hydrogels, self-healing polymer networks, bioactive vitreous replacements, in situ crosslinked hyaluronic acid-based systems, and standardized in vitro platforms for assessing rheology and diffusion.

The final reference selection was organized to support a coherent, mechanism-based narrative. The aim was to trace the movement of the field from purely mechanical tamponades toward bioengineered materials that may better approximate the viscoelastic and biochemical roles of the native vitreous.

The author DR declares that generative AI tools were used during manuscript preparation for figure and table drafting support, proofreading, and language polishing.

## 2. Native Vitreous: Structure, Mechanics, and Biochemical Functions

The vitreous is a very soft extracellular matrix, composed of more than 98% water retained within a network of collagens, hyaluronic acid, proteoglycans, and soluble proteins [1,15]. Collagen types II, IX, and XI form heterotypic fibrils that provide elastic support: type IX helps stabilize fibril spacing, whereas type XI contributes to fibril diameter. Non-collagenous components are also essential. Opticin, the predominant vitreous leucine-rich repeat protein, binds selectively to collagen fibrils and helps regulate fibril interactions, thereby contributing to the optical and biomechanical clarity of the vitreous [17,18]. Its anti-adhesive properties may limit abnormal vitreoretinal adhesion, while its inhibition of endothelial migration and tube formation supports the anti-angiogenic character of the normal vitreous [19,20]. Hyaluronic acid occupies the interfibrillar space and retains water, while proteoglycans help regulate fibril spacing and mechanical stability. Hyalocytes add another layer of biological activity through extracellular-matrix turnover and immune modulation [21]. Proteomic and transcriptomic studies have identified more than 2000 vitreous proteins, including growth factors, metalloproteinases, antioxidant enzymes, and complement components, confirming that the vitreous is an active biochemical interface between the retina and the anterior segment [22]. In chemical terms, type II collagen is the dominant fibril-forming collagen of the vitreous and provides the triple-helical backbone of the fibrillar scaffold. Type IX collagen is a fibril-associated collagen with interrupted triple helices; its non-collagenous domains and glycosaminoglycan substitutions project from the fibril surface and help maintain spacing and hydration. Type XI collagen is incorporated within heterotypic fibrils and regulates nucleation and fibril diameter. These differences explain why the three collagen types are not interchangeable: type II mainly provides tensile architecture, type IX stabilizes fibril surface interactions and spacing, and type XI limits excessive fibril growth, preserving transparency and soft mechanics [17,18].

**Figure 1:**
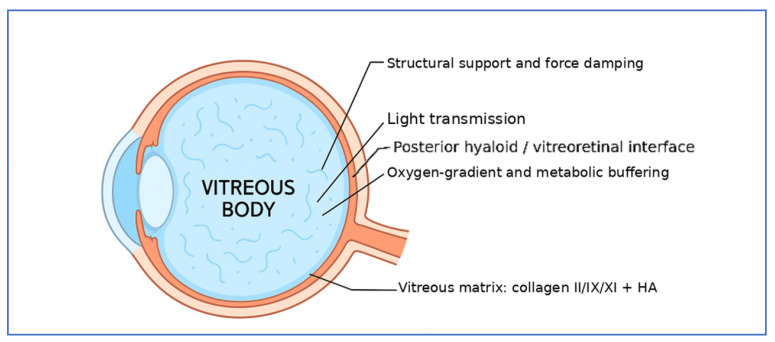
Structure and physiological functions of the native vitreous body. The illustration shows a cross-section of the human eye and distinguishes the vitreous matrix (collagen types II/IX/XI interwoven with hyaluronic acid, HA) from the posterior hyaloid and vitreoretinal interface. The collagen-HA network contributes to structural support, force damping, optical transparency, oxygen-gradient regulation, metabolic exchange, and buffering of reactive oxygen species. These integrated properties help preserve retinal function, optical clarity, and intraocular homeostasis.

Mechanically, the vitreous behaves as a viscoelastic material. It combines a weak solid-like elasticity with fluid-like viscosity, allowing collagen microfibrils, hyaluronic acid, and water to work together in absorbing mechanical stress, maintaining ocular shape, stabilizing intraocular pressure, and supporting the retina [8,23]. Its refractive index, approximately 1.3345, is close to that of adjacent ocular media, which helps minimize light scattering. Rheological measurements indicate a zero-shear viscosity of 300–2000 mPa·s and a storage modulus of 0.05–2 Pa, values that place the vitreous among the softest connective tissues [8,24]. Its gel structure also restricts convective flow and favors diffusion-mediated exchange of oxygen and metabolites, thereby acting as a biochemical buffer against local hypoxia or oxidative stress [5,15]. Stefánsson [4] showed that the intact vitreous limits oxygen flux toward the lens and may protect against nuclear cataract formation; more recent work has also emphasized how oxidative imbalance and antioxidant depletion increase lens vulnerability after vitrectomy [25]. These findings help explain why the vitreous should be regarded as both a mechanical and a protective tissue (Figure 1).

Aging gradually disrupts this organization. Hyaluronan integrity declines, while collagen undergoes oxidative modification and proteolytic cleavage. The result is fibril aggregation, phase separation or synchisis, and collapse of residual fibrils, known as syneresis. Clinically, these changes appear as liquefaction, light scattering, and lacunae that may be detected by optical coherence tomography (OCT) or ultrasonography [23,26]. Weakening of the posterior vitreous cortex can promote posterior vitreous detachment (PVD). Although PVD is often physiological, it may predispose susceptible eyes to retinal tears, detachment, or macular traction. Proteomic studies also show increased oxidative stress, adhesion-molecule remodeling, and extracellular-matrix alterations, suggesting that reactive oxygen species may degrade hyaluronan, alter collagen cross-links, disturb gel viscosity, and activate matrix metalloproteinases [22,27].

Several ocular and systemic conditions can accelerate these processes. In diabetes, hyperglycemia-induced oxidative stress and advanced glycation end-products alter the vitreous matrix, reduce permeability, increase liquefaction, accelerate PVD, and contribute to fibrovascular proliferation in proliferative diabetic retinopathy [7,28]. High myopia is associated with earlier vitreous liquefaction and PVD. In addition, proteomic and cytokine studies have reported increased pro-inflammatory and angiogenic mediators in highly myopic or inflamed eyes, creating a milieu that favors abnormal remodeling and tractional complications [29,30,31,32]. Glycation, oxidative stress, and cytokine signaling therefore appear to converge in the degeneration of the vitreous matrix [27].

These structural and biochemical changes are relevant not only for understanding age-related and disease-related vitreoretinal disorders. They also set the design criteria for a replacement material. A substitute that only occupies space will not address the loss of viscoelastic support, diffusion control, and oxidative-buffering capacity that normally belong to the vitreous [8,12].

## 3. Functional Requirements for Vitreous Replacement

The functional deficit left by vitrectomy explains why the requirements for an ideal vitreous substitute are so demanding. A successful replacement cannot simply fill the cavity. It should reproduce, as much as realistically possible, the optical, mechanical, and biological roles of the native vitreous. Recent reviews on hydrogel-based substitutes make this point clearly: the material should be transparent, soft, hydrated, stable in the posterior segment, and biologically tolerated, without causing inflammation, toxicity, excessive degradation, or optical disturbance [33]. To make these requirements less qualitative, Table 1 summarizes indicative target ranges that are commonly used as design benchmarks rather than absolute clinical thresholds.

Optical compatibility is the first practical requirement. A vitreous substitute must remain highly transparent across the visible spectrum and should have a refractive index close to that of the native vitreous, about 1.3345. Otherwise, it may cause light scattering, image distortion, or clinically relevant refractive shifts. Even small degrees of opacification, phase separation, or particle formation matter because the material lies directly in the visual axis. For this reason, clarity should be evaluated not only immediately after injection, but also after time, mechanical stress, enzymatic exposure, and contact with intraocular proteins.

The second requirement is viscoelasticity. The native vitreous is not a simple fluid. It behaves as a very soft gel, combining elastic support with viscous damping. This allows it to absorb ocular movement, distribute mechanical forces, and support the retina gently, without transmitting excessive shear stress to fragile posterior-segment tissues. A substitute should therefore approach the rheological behavior of natural vitreous. If it is too fluid, it may not provide stable support; if it is too stiff, it may interfere with ocular biomechanics or increase traction at the vitreoretinal interface [8,33].

Biocompatibility is equally decisive. The material must be non-toxic and non-immunogenic, and it must be compatible with retinal neurons, Müller cells, retinal pigment epithelium, hyalocytes, vascular structures, and the trabecular outflow pathway. Clinical experience with current tamponades has shown that even materials considered relatively inert may cause problems when they persist in the eye, including inflammation, emulsification, secondary glaucoma, keratopathy, retinal toxicity, and the need for later removal [5,12]. For new substitutes, both the intact material and its degradation products must therefore be tested carefully. Long-term stability may be useful in some indications, whereas predictable degradation may be preferable in others, but neither should lead to fragmentation, inflammatory activation, or accumulation in anterior-chamber structures.

A further requirement is preservation of a tolerable intravitreal microenvironment. The native vitreous contributes to oxygen-gradient regulation, metabolite diffusion, cytokine movement, and oxidative balance. After vitrectomy, this regulatory function is altered, and increased oxygen exposure to the lens and changes in posterior-segment homeostasis may contribute to cataract progression and other postoperative complications [5,25]. An ideal substitute should therefore allow controlled diffusion of oxygen, nutrients, metabolites, and therapeutic molecules, while avoiding excessive convective flow. For long-term replacement, some capacity to buffer oxidative stress or support redox homeostasis would be especially valuable. This point is distinct from disease-specific bioactivity. Optical transparency, refractive compatibility, injectability, soft viscoelasticity, controlled swelling, and biocompatibility are general requirements for any long-residence substitute. Antioxidant, anti-inflammatory, anti-VEGF, NRF2-activating, or anti-fibrotic functions are optional additions that should be matched to specific indications such as proliferative vitreoretinopathy, proliferative diabetic retinopathy, chronic inflammation, or postoperative scarring.

The oxygen issue is particularly relevant for the crystalline lens. After vitrectomy, convection and oxygen transport from the retina toward the anterior segment increase, and the loss of the native gel removes part of the diffusion barrier that normally helps maintain a lower oxygen tension near the posterior lens surface. Gas bubbles and balanced salt solution provide little long-term buffering. Conventional silicone oil can modify intraocular oxygen distribution by creating an immiscible phase, but it does not restore physiological diffusion and may be associated with anterior-segment complications. A hydrogel substitute with native-like diffusivity could, in principle, reduce excessive oxygen delivery to the lens while still allowing metabolic exchange; however, this cataract-protective effect remains a target for validation rather than a demonstrated clinical benefit [4,25].

In practical terms, the desired material profile is demanding: optical clarity, refractive compatibility, soft but stable mechanics, injectability through small-gauge systems, biological tolerance, controlled swelling or degradation, and preservation of diffusion-mediated retinal homeostasis. This profile also explains why current clinical options remain incomplete. Gases, silicone oils, and perfluorocarbon liquids are surgically useful, but they do not reproduce the viscoelastic, diffusional, and biochemical roles of the vitreous. The challenge is therefore to move from passive intraocular fillers toward biomimetic materials able to restore both structural support and homeostatic function.

## 4. Current Clinical Vitreous Substitutes and Their Limitations

Intraocular gases were among the first widely adopted vitreous substitutes because their surface tension can close retinal breaks and provide short-term macular or retinal support. Air disappears rapidly, whereas expansile gases such as sulfur hexafluoride (SF_6_) and octafluoropropane (C_3_F_8_) last longer because they expand predictably and are gradually resorbed [34]. Their action, however, is almost entirely mechanical. They do not reproduce vitreous-like viscoelasticity, diffusion control, or oxidative buffering, and therefore cannot restore metabolic homeostasis in the postoperative posterior segment [12]. This is why they remain useful but incomplete tools in vitreoretinal surgery.

**Figure 2:**
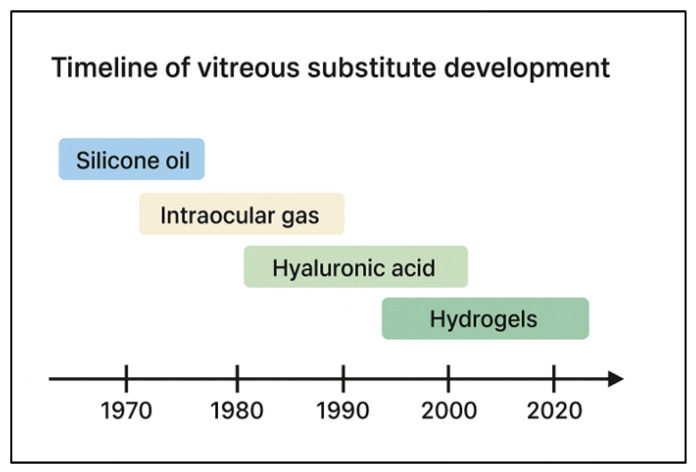
Timeline of vitreous substitute development. The scheme summarizes the evolution of vitreous substitutes from the 1970s to the present. Silicone oils were introduced as long-lasting tamponade agents, followed by expansile intraocular gases for temporary support. Hyaluronic acid-based viscoelastic agents entered clinical evaluation in the late 1980s and 1990s. Since the early 2000s, hydrogel-based biomaterials have become leading candidates for next-generation vitreous replacement.

Silicone oils offered a more durable form of tamponade, capable of supporting retinal attachment for longer periods after surgery. Their behavior depends on low specific gravity, high interfacial tension, and relative chemical inertness, which allow them to remain in the vitreous cavity as long-lasting fillers [35]. Yet the clinical experience accumulated over decades shows that their intraocular presence is not biologically neutral. Emulsification is the major limitation. Ocular movements and pulsation fragment the oil into microdroplets, which then interact with proteins, lipids, and inflammatory mediators. These droplets may enter the anterior chamber, obstruct the trabecular meshwork, or spread along retinal surfaces. The process is multifactorial and is associated with secondary glaucoma, keratopathy, and chronic inflammation [36,37]. Case series and imaging studies also indicate that prolonged silicone-oil exposure may induce inner retinal toxicity and structural changes that can persist even after removal [38,39,40]. Although changes in viscosity and formulation have been explored, they have not removed the fundamental problem: silicone oil does not mimic the viscoelastic, diffusional, or biochemical functions of the native vitreous [41]. A more detailed comparison is useful clinically. Standard 1000-cSt silicone oil is easier to inject and remove but is generally more prone to emulsification than higher-viscosity formulations. High-viscosity oils, such as 5000-cSt oil, may reduce droplet formation but are more difficult to manipulate through small-gauge systems and still require removal when complications or optical disturbance occur. Heavy silicone oils contain fluorinated or semi-fluorinated components to increase density and improve inferior tamponade; however, these formulations may show greater emulsification, inflammatory reactivity, and interface toxicity than standard oils. Semi-fluorinated alkanes can improve solubility and density tuning, but incomplete mixing, degradation, or residual droplets may create additional safety concerns. Thus, the choice among conventional, high-viscosity, heavy, and semi-fluorinated systems is a compromise between surgical geometry, inferior retinal support, ease of handling, emulsification risk, and tolerance of prolonged intraocular residence [35,41,42,43] (Figure 2).

Heavy silicone oils and fluorinated compounds were introduced to improve tamponade of inferior retinal pathology. Their higher density can increase contact with the lower retina, where conventional oils are less effective. At the same time, however, density changes may alter fluid shear and increase tissue-interface interactions, with higher risks of emulsification, inflammation, and difficult removal. Fluorinated additives make density adjustment possible, but they may also introduce cytotoxicity or accelerated degradation. For these reasons, heavy oils are generally reserved for selected complex cases, where their mechanical advantage must be weighed against their greater in vivo reactivity and the frequent need for early removal [42,43].

Perfluorocarbon liquids show the same trade-off even more clearly. In the operating room, they are extremely valuable: their high specific gravity and low viscosity help flatten detached retinas, displace subretinal fluid, and stabilize mobile retinal flaps with great precision. These properties depend on the carbon-fluorine backbone and low interfacial reactivity. However, they are not suitable for long-term residence. Retained droplets may remain on the retinal surface, trigger macrophage responses, cause localized toxicity, and contribute to proliferative vitreoretinopathy. Recent scoping reviews confirm both the surgical usefulness of PFCLs and the concern over complications caused by even small residual volumes, especially in severe trauma or complex retinal reattachment [44]. Techniques such as drip-irrigative clearance have been proposed to improve removal, but incomplete clearance remains possible. PFCLs should therefore be regarded as short-term surgical aids, not long-term vitreous substitutes [45].

Balanced salt solutions and similar aqueous irrigants have a much more limited role. They maintain intraocular volume and chamber stability during surgery, but they behave very differently from viscoelastic materials. They provide no structural support, no cushioning effect, and no relevant biochemical protection beyond short-term space maintenance. Studies of BSS-assisted intraocular lens implantation show that BSS can maintain chamber depth, but lacks the viscoelastic and endothelial-protective properties of conventional ophthalmic viscoelastic devices [46,47]. In other words, BSS is useful during surgery, but it has no realistic role as a mechanical or metabolic vitreous substitute.

From a biomaterials perspective, the shared weakness of current substitutes is that they do not truly engage with the ocular environment. Gases and oils act through interfacial mechanics, buoyancy, surface tension, or density. They do not reproduce the heterogeneous collagen-hyaluronan architecture of the native vitreous. They also do not buffer reactive oxygen species, modulate cytokine diffusion, or maintain the oxygen gradients that help protect ocular tissues [4]. This gap has become more apparent as proteomic and structural studies have recast the vitreous as a dynamic soft material rather than a passive filler [8,22]. Incremental improvements in oils and PFCL handling may improve safety, but current tamponades still depend mainly on mechanical support, postoperative positioning, or secondary removal [16]. Meaningful progress is therefore likely to come from engineered hydrogels, peptide-based materials, and multifunctional polymers that can better approximate the compliance, hydration, diffusion behavior, and biological buffering of the natural vitreous (Table 2).

## 5. Emerging Hydrogel and Polymer-Based Vitreous Substitutes

Because conventional tamponades have clear limitations, much attention has shifted to bioengineered hydrogels [8,13,33]. This shift is understandable. Hydrogels are water-rich three-dimensional polymer networks, and their basic structure makes them closer to the native vitreous than gases or oils. They can be designed to be transparent, soft, injectable, and mechanically tunable. Some can also be engineered to provide biochemical or drug-delivery functions. This does not mean that hydrogels are already a solved clinical solution, but they currently offer the most plausible route toward a more physiological vitreous replacement.

### 5.1. Natural Polymer-Based Systems

Hyaluronic acid-based systems have provided some of the earliest translational signals in this field. In patients with phthisis bulbi, cross-linked hyaluronic acid hydrogels have been used intraocularly to help support globe integrity and stabilize intraocular pressure [48]. This experience is relevant, but it should be interpreted cautiously. Phthisis bulbi is a very specific setting, and success in that context does not prove that the same materials can safely replace the vitreous in functional eyes over long periods. In parallel, bioinspired thermosensitive hydrogels have shown how an injectable polymer can be delivered as a fluid and then gel under physiological conditions [49].

Among current approaches, in situ crosslinked hyaluronic acid-based hydrogels are particularly attractive because they combine a native-matrix component with injectability, controlled network formation, and compatibility with small-gauge vitreoretinal surgery [13]. Their performance, however, depends strongly on polymer source and crosslinking chemistry. Gelation strategy, swelling behavior, enzymatic susceptibility, and post-injection stability are not minor technical details. They are among the variables that determine whether a material can function inside the eye rather than only look promising in vitro [13,50]. Thermoresponsive transitions, photocrosslinking, enzymatic crosslinking, and reversible dynamic bonds all offer possible routes to injection through small-gauge cannulas followed by gel formation in vivo [49,51].

Despite these advantages, HA-based systems also have important limitations. Native HA is susceptible to hyaluronidase-mediated degradation, and chemical modification or crosslinking is needed to prolong residence. Excessive crosslinking, however, can reduce injectability or alter biocompatibility. Swelling is another critical issue: a hydrogel that expands unpredictably after injection may increase intraocular pressure, compress retinal tissues, or migrate anteriorly. Mechanical durability is also not guaranteed, because repeated ocular movement may fatigue weak networks and produce fragments that could scatter light or obstruct outflow. For this reason, HA-based substitutes should be discussed as promising but not intrinsically safe or mechanically sufficient; their performance depends on molecular weight, crosslinking chemistry, swelling control, enzymatic stability, and degradation products [13,50,52].

Natural polymer-based hydrogels remain appealing because they are generally biocompatible and resemble elements of the ocular extracellular matrix. Self-assembling peptide hydrogels, for example, form ECM-like nanofibrillar structures and have shown good transparency and retinal compatibility in rabbit models [53]. Hyaluronic acid, a native vitreous component, has been chemically modified to increase enzymatic stability and residence time while preserving optical clarity [52,54]. Recent work also shows that HA-based hydrogels can be tuned for clinically relevant properties, including swelling control, enzymatic degradation, and cellular adhesion; UV-crosslinked HA formulations, for instance, show format-dependent swelling, selective hyaluronidase-mediated degradation, and reduced fibroblast proliferation on the hydrogel surface [50]. Other natural or semi-natural systems broaden the field further. Gelatin methacryloyl (GelMA) provides a photopolymerizable matrix with retinal tissue compatibility [55], alginate-HA blends show promising in vitro viscoelastic and optical properties [56], and self-healing chitosan-HA hydrogels combine injectability with retinal biocompatibility in preclinical models [57].

### 5.2. Synthetic Polymer-Based Systems

Synthetic hydrogels provide more precise control over network architecture, degradation kinetics, and optical behavior. Poly(ethylene glycol) (PEG) is widely used because it is hydrophilic, chemically versatile, and suitable for controlled crosslinking [58]. Thermoresponsive synthetic systems are also advancing. PNIPAAm-based and poloxamer-based hydrogels can gel at body temperature and, in some formulations, serve as sustained intravitreal drug-delivery platforms. A key experimental study showed that a thermogelling polymer endotamponade could reattach the retina and partially re-form a vitreous-like body in rabbits and non-human primates [59]. More recently, a nano-enabled thermoresponsive hydrogel provided prolonged triamcinolone acetonide release after fine-gauge injection, with evidence of ocular safety and controlled pharmacologic activity in vivo [60].

### 5.3. Bioactive and Drug-Delivering Hydrogels

The most interesting direction, in our view, is the development of hydrogels that do more than occupy space. Oxidative stress is involved in several postsurgical complications, including proliferative vitreoretinopathy, and antioxidant-releasing hydrogels have therefore been designed to protect retinal cells by scavenging reactive oxygen species. A PEG-based hydrogel containing vitamin C provided continuous radical neutralization while maintaining structural integrity, illustrating how structural support and therapeutic function may be combined [58]. Other multifunctional hydrogels are being explored for sustained release of corticosteroids, antiproliferative agents, or anti-VEGF molecules [60]. For these systems, however, drug loading and release duration are not enough. The material must also reproduce, or at least not distort, physiologically meaningful intravitreal diffusion. In one recent in vitro study, one-component gels such as single-agent HA, hypromellose, and polyacrylamide did not reproduce both rheological similarity and vitreous-like diffusion, whereas two-component non-covalent HA-agar hydrogels more closely matched porcine vitreous diffusion of triamcinolone acetonide [61]. EGCG-loaded HA hydrogels also illustrate this direction, combining antioxidant activity with good biocompatibility for vitreous replacement [54].

**Figure 3:**
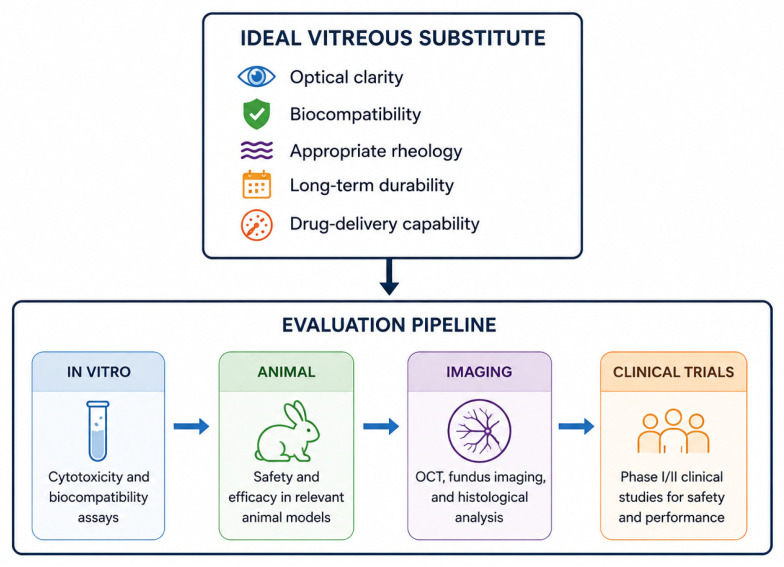
Schematic of ideal properties and the preclinical evaluation pipeline for next-generation vitreous substitutes. The flowchart summarizes the main physicochemical and biological requirements for an ideal vitreous substitute: optical clarity, physiological viscoelasticity, biocompatibility, long-term stability, and potential drug-delivery capability. It also outlines the corresponding evaluation pathway, including in vitro cytotoxicity and oxidative-stress assays, small- and large-animal studies for biocompatibility and mechanical endurance, optical and rheological characterization, multimodal imaging with OCT and fundus examination, histopathology, and eventual progression to early-phase clinical studies.

Mechanistically, antioxidant hydrogels may act in several ways. Vitamin C- or polyphenol-loaded networks can directly neutralize free radicals and reduce lipid peroxidation, while sustained local release may replenish part of the antioxidant buffering lost with native vitreous removal. EGCG-containing HA hydrogels add phenolic radical-scavenging capacity and may also attenuate inflammatory and pro-angiogenic signaling. NRF2-activating polymers act differently: rather than only consuming ROS, they stimulate endogenous cytoprotective transcriptional programs, including heme oxygenase-1, glutathione-related enzymes, and other phase-II antioxidant responses. Anti-fibrotic effects are most relevant to PVR-like biology, where oxidative stress, macrophage activation, Müller-cell gliosis, RPE migration, myofibroblast differentiation, TGF-beta signaling, extracellular-matrix deposition, and contractile membrane formation interact. A biofunctional gel that reduces oxidative injury and suppresses scarring pathways may therefore be useful in selected high-risk eyes, but it should not be considered a universal requirement for every vitreous replacement [54,58,62] (Figure 3).

Beyond ROS scavenging, some next-generation hydrogels are being designed to interfere with fibrotic signaling. A recent polymer platform, for example, activated NRF2-mediated cytoprotection and suppressed retinal scarring, suggesting that a vitreous substitute could eventually act as both a scaffold and a local modulator of disease pathways [62].

### 5.4. Hybrid and Composite Systems

No single polymer architecture is likely to reproduce all mechanical and biochemical functions of the vitreous. For this reason, hybrid and composite systems are increasingly attractive. Interpenetrating networks that combine hyaluronic acid with PEG, or nanocomposites that reinforce GelMA with cellulose-based nanofibers, may improve mechanical resilience, slow degradation, and enhance cytocompatibility compared with single-component systems [51]. These composite strategies are not merely material refinements. They reflect the central challenge of the field: integrating transparency, viscoelasticity, antioxidant capacity, controlled diffusion, and long-term tissue compatibility into one material that can realistically be used inside the human eye.

## 6. Preclinical Evaluation and Biocompatibility Assessment

Preclinical testing is a critical step for any vitreous substitute intended for human use. The posterior segment tolerates only a narrow range of mechanical, chemical, and oxidative disturbances, so candidate materials must be evaluated for cytotoxicity, immunogenicity, and effects on retinal physiology. Early work usually begins with in vitro models, including retinal pigment epithelial cells, Müller glia, and photoreceptor-derived lines. These assays can detect acute cytotoxicity, mitochondrial dysfunction, and pro-inflammatory signaling triggered by the material or by its degradation products. Oxidative-stress measurements, such as ROS generation and glutathione depletion, are also important. A material that releases radicals, accumulates oxidants, or fails to buffer oxidative stress may accelerate gliosis and photoreceptor injury. Conversely, antioxidant-releasing hydrogels have shown reduced ROS generation and favorable cell viability in such models [54,58]. Long-residence materials should also be tested for leachable monomers and crosslinker fragments that could disturb retinal metabolism or activate complement pathways.

Safety-oriented design is becoming more sophisticated. Some hydrogels now incorporate anti-fouling and anti-proliferative properties to reduce postoperative tissue reactions. Dual-crosslinked amphiphilic systems, for example, show strong anti-adhesion behavior, less protein deposition, and reduced abnormal cell proliferation. These properties may help limit inflammation, fibrocellular membrane formation, and optical haze while preserving intravitreal stability [63].

Promising formulations then move into animal models chosen for anatomical and physiological relevance. Rabbits remain the usual first in vivo model because they are manageable, their surgical protocols are well established, and the vitreous cavity is large enough to evaluate injection, distribution, and medium-term residence. Rabbit studies have supported the feasibility and biocompatibility of several in situ-forming gels, including thiol-crosslinked hyaluronan derivatives and self-assembling peptide systems, which remained optically clear and preserved retinal morphology over weeks [53,64,65]. Porcine eyes are useful at a later stage because their vitreous volume and vitreoretinal interface more closely resemble those of human eyes; they are therefore valuable for testing rheological stability, gel-tissue interaction, and mechanical endurance under ocular movement [66]. Each model also has limitations that should be made explicit. Rabbit eyes are convenient and sensitive for first safety screening, but their vitreous volume, lens size, ocular growth pattern, and inflammatory response differ from those of adult human eyes; therefore, a gel that appears stable in rabbits may not predict long-term human performance. Porcine eyes are closer to human eyes in globe size and are useful for rheology, injection, and diffusion studies, but most porcine work is ex vivo or short-term and cannot fully address chronic inflammation, cataractogenesis, or immune response. Non-human primates provide the most relevant anatomy and visual system, but their use is limited by cost, ethics, small sample size, and restricted follow-up; they should therefore be reserved for questions that cannot be answered in lower models. These limitations support a staged approach rather than reliance on a single species [59,66,67].

Non-human primates are used only when the translational question justifies it, but they provide the closest approximation to human immunologic and visual-function responses. A thermogelling polymer endotamponade, for instance, showed stable long-term residence and retinal compatibility in cynomolgus monkeys [59]. More generally, advanced in vivo studies indicate that candidate substitutes should be judged not only by short-term anatomical tolerance, but also by persistence, inflammatory response, rheological stability, drug-delivery performance, and suitability for long-term intraocular use [52,60]. Across models, the essential readouts are clear gel residence, lack of chronic anterior or posterior inflammation, preserved retinal architecture on imaging, and maintenance of electrophysiological function (Table 3).

Physicochemical testing is just as important as biological observation. Rheometry provides storage (G′) and loss (G″) moduli and helps define the viscoelastic profile of the material. A plausible substitute should fall within, or at least near, the rheological envelope of native human or porcine vitreous. Oscillatory shear, creep-recovery, and large-strain testing show whether a gel can resist mechanical collapse during ocular motion without becoming so stiff that it transmits damaging shear to the retina. Recent comparative studies suggest that some advanced composite and synthetic hydrogels can approach native rheological behavior, even after accelerated aging [51,68]. Optical testing is equally necessary. Spectrophotometry should show high visible-spectrum transmittance, ideally at least 90%, and refractometry should confirm values close to the physiological refractive index, about 1.334–1.336. Finally, rheology alone is not enough. Diffusion-benchmarking studies indicate that substitutes should also be compared with native or porcine vitreous for drug diffusion, dissolution behavior, and stability under standardized in vitro conditions [61].

Noninvasive imaging and histopathology help connect material performance with retinal safety. OCT allows longitudinal monitoring of the vitreoretinal interface, gel residence, retinal thickness, and early signs of separation or migration [69]. Fundus photography and wide-field imaging can document inflammation and perfusion, while fluorescein or indocyanine green angiography may reveal subtler vascular compromise. Histopathology remains the final check. Semithin and ultrathin sections of the retina, RPE, choroid, and optic nerve can reveal photoreceptor outer-segment damage, Müller cell gliosis, microglial infiltration, or foreign-body reaction at the gel interface. Correlations between OCT and histology in preclinical ocular safety studies support the value of noninvasive imaging for major adverse events, although ultrastructural abnormalities may still require electron microscopy or immunohistochemistry [67,70].

A rigorous preclinical pipeline should therefore combine in vitro cytotoxicity and oxidative-stress testing, small-animal safety and residence studies, larger-animal mechanical endurance and function-preserving evaluations, and final multimodal imaging with histology to exclude late toxicity. Translational studies show that this staged approach can identify materials with acceptable rheology, clarity, biological tolerance, and therapeutic performance before clinical evaluation [52,58,60]. Even so, longer surveillance and more harmonized cross-laboratory methods are still needed. Subtle neurotoxicity or chronic low-grade inflammation may not appear in short studies. Standardized testing should therefore include not only cytotoxicity, imaging, and histopathology, but also swelling, enzymatic degradation, cell adhesion, drug diffusion, and post-injection rheological stability [50,61].

## 7. Translational Challenges and Early Clinical Experience

Moving advanced vitreous substitutes into the clinic will require more than promising preclinical data. This point deserves emphasis because the posterior segment is a delicate and visually critical space. A material that performs well in a rheometer, a cell-culture system, or even a short animal study may still behave unpredictably after months inside the eye. At present, clinical experience with hydrogel-based vitreous replacements remains very limited. One of the few examples is a retrospective interventional study in phthisis bulbi, where a UV-cross-linked hyaluronan gel was used to help stabilize intraocular pressure and preserve retinal structure over weeks to months [48]. This is useful evidence, but it does not yet establish a polymer-based substitute for long-term use in functional, ambulatory eyes.

Translation is slow partly because the regulatory burden is high, and appropriately so. Manufacturing must meet drug- or device-level Good Manufacturing Practice standards, and the material must satisfy biocompatibility requirements such as those in the ISO 10993 series. Regulators may also draw on established ophthalmic-device standards, including the ISO 11979 series for intraocular lenses, when considering novel vitreous implants [52]. Recent reviews of HA-based in situ injectable hydrogels emphasize the same point: translation will depend on process reproducibility, scalable manufacturing, standardized in vitro workflows, physiologically relevant eye models, and clearer regulatory benchmarks for materials that remain in the vitreous cavity [13]. Before first-in-human studies, developers must therefore demonstrate long-term preclinical safety, chronic inflammation surveillance, manufacturing validation, and reproducible physicochemical and diffusion testing.

Even after early human use becomes possible, complications must be expected and actively monitored. Cross-linked hyaluronic acid hydrogels such as Healaflow have been tested in rabbit eyes, where loss of gel viscosity and some cataract formation were observed [71,72]. In compassionate-use phthisis-bulbi patients, intraocular-pressure stabilization and retinal structural preservation were reported, but mild inflammatory changes and variable gel persistence were also noted [52]. Inflammation, opacification, or gel degradation may disturb visual quality. Secondary elevation of intraocular pressure, already familiar from traditional tamponades, may also occur. Degradable or non-resorbable polymer fragments could accumulate in anterior structures, affecting trabecular outflow or causing toxicity. For HA-based systems, swelling after injection, hyaluronidase-mediated degradation, and interaction with fibroblastic cells relevant to proliferative vitreoretinopathy should be treated as clinically meaningful risk variables, not as secondary laboratory details [50].

Future early-phase trials should therefore monitor more than simple anatomical tolerance. Visual acuity, OCT anatomy, intraocular pressure, electrophysiology, patient-reported visual quality, inflammation, and material degradation all need structured follow-up. A short-term absence of obvious toxicity should not be considered sufficient evidence of success. Regulatory progress will require open dialogue among surgeons, material scientists, manufacturers, and health authorities, so that meaningful endpoints, manufacturing controls, and appropriate follow-up durations can be agreed upon. Only through this staged and rather cautious development can hydrogel vitreous substitutes move from promising experimental constructs to safe and useful clinical tools.

## 8. Future Directions in Vitreous Substitution

Materials science, regenerative medicine, and computational design are now pushing vitreous substitution away from static fillers and toward dynamic systems. The next generation of injectable materials is likely to include shear-thinning, self-healing, stimuli-responsive, and in situ crosslinking polymers. These systems can pass through fine-gauge cannulas and then recover mechanical integrity inside the eye. Dual-crosslinked amphiphilic materials, for example, combine tunable viscoelasticity with self-healing and anti-fouling behavior, reducing protein deposition and surface adhesion while maintaining optical clarity [51,63]. Thermogelling polymers remain a major focus, especially after in vivo studies showing re-formation of a vitreous-like body and effective endotamponade in rabbits and primates [59], alongside broader interest in thermogels for ocular applications [73,74]. HA-based in situ crosslinked systems are also especially promising because they build on a native vitreous component while allowing control over gelation, swelling, degradation, and injectability [13,50].

The future challenge is not only better mechanics. It is also biological function. Biofunctional substitutes are being designed to influence the biochemical environment of the posterior segment. Antioxidant-releasing hydrogels provided an early proof of concept, and newer materials are being explored for direct modulation of cellular pathways involved in fibrosis. Polymers that activate endogenous cytoprotective pathways such as NRF2, for example, may help suppress fibrotic cascades and limit tractional recurrence [62]. Sustained release of anti-inflammatory, anti-fibrotic, or anti-VEGF agents could also make the substitute part of a local therapeutic strategy rather than a passive filler. At the same time, the material must be treated as a diffusion-regulating environment. Recent in vitro work showed that hydrogels with acceptable rheological features may still fail to reproduce physiologically relevant drug diffusion, whereas two-component HA-agar hydrogels more closely matched triamcinolone acetonide diffusion in porcine vitreous [61]. Future candidates should therefore be optimized through integrated testing of transparency, viscoelasticity, swelling, degradation, cellular response, and molecular transport.

A more ambitious possibility is the development of bioprinted or cell-supportive vitreous analogues. These materials could serve both as structural matrices and as permissive scaffolds for regenerative approaches. Self-assembling peptide hydrogels already provide nanofibrillar networks that are attractive for this purpose [53]. In combination with HA- and GelMA-based bioinks and progress in retinal tissue engineering, future substitutes might provide trophic support, allow controlled cell delivery, or create a more favorable microenvironment for photoreceptor integration [75]. These applications remain speculative, but they show how vitreous substitution may eventually overlap with retinal regenerative medicine.

Computational methods may also accelerate material development. Machine learning models and multiphysics simulations can help predict viscoelasticity, degradation kinetics, and oxygen diffusion before a polymer is synthesized. This does not replace experimental validation, but it can reduce empirical trial and error and help prioritize promising formulations. Finite-element and transport models may be particularly useful for anticipating long-term behavior under ocular motion [76,77]. In this sense, artificial intelligence is best viewed as a design and optimization tool for complex hydrogel systems, not as a substitute for biological testing.

Looking further ahead, some vitreous substitutes may eventually combine mechanical replacement with bioelectronic or prosthetic functions. Hybrid materials containing microscale reservoirs, conductive components, or electrode-compatible structures could, in principle, stabilize the retina while supporting controlled neuromodulation. This remains a distant concept, but progress in biodegradable electronics and conductive hydrogels makes it a plausible area for future exploration.

The direction of the field is therefore clear, even if clinical translation remains difficult. Vitreous replacement is moving from inert tamponades toward multifunctional and biologically interactive materials. To make this transition clinically credible, the field will need rigorous preclinical pipelines, AI-assisted but experimentally validated material design, scalable manufacturing, and early human studies that assess both mechanical efficacy and true biological benefit (Table 4).

## 9. Conclusions

Vitreoretinal surgery has advanced enormously, but the absence of a true vitreous substitute remains a basic limitation. Current tamponade agents, including gases, silicone oils, and perfluorocarbon liquids, are indispensable in daily practice. They help surgeons solve difficult anatomical problems. Still, they act mainly as mechanical tools and do not restore the physiological roles of the native vitreous. In many cases, anatomical success can be achieved, but the postoperative eye is left in a biological environment that is still far from normal.

This is where biomaterials science becomes essential. Bioengineered hydrogels and multifunctional polymer systems may eventually restore not only intraocular volume and retinal support, but also viscoelastic damping, controlled diffusion, regulated swelling and degradation, and protection against oxidative or inflammatory stress. The most convincing future substitutes will probably be those that combine native-like mechanics with predictable degradation, controlled molecular transport, and favorable cellular interactions. At the same time, the field should avoid premature enthusiasm. A material that is transparent, injectable, and well tolerated in early testing is not automatically a physiological vitreous substitute. Long-term safety, diffusion behavior, inflammatory response, manufacturability, and regulatory reproducibility will determine whether these materials can truly enter clinical practice.

The movement from inert tamponades toward biologically interactive substitutes is nevertheless an important conceptual step. Progress will depend on careful preclinical validation, cautious early-phase trials, and close collaboration among surgeons, material scientists, manufacturers, and regulatory bodies. The final goal is not simply to fill the vitreous cavity. It is to rebuild, as far as possible, an intraocular environment that can support durable retinal health and visual function.

## Figures and Tables

**Table 1:** Indicative quantitative design targets for long-term vitreous replacement. The ranges are approximate and should be interpreted in relation to the indication, residence time, degradation profile, and testing method [8,15,24,33].

Parameter	Indicative Target Range or Direction	Rationale and Testing Considerations
Refractive index	Approximately 1.334–1.336	Close to native vitreous and aqueous media to avoid refractive shift or image distortion.
Visible-light transmittance	At least 90%, preferably >95% across the visible range	Maintains optical clarity after injection, aging, protein exposure, and mechanical stress.
Storage modulus (G′)	Approximately 0.05–2 Pa	Approaches the very soft solid-like component of native vitreous without adding tractional stress.
Zero-shear viscosity	Approximately 300–2000 mPa·s	Provides damping and diffusion control while preserving injectability and surgical handling.
Swelling ratio/volume change	Minimal; ideally <5–10% net volume expansion in physiological media	Limits IOP elevation, retinal compression, and anterior migration after injection.
Degradation or residence time	Indication-dependent; temporary: weeks-months or long-term >6–12 months without fragmentation	Should match the surgical goal and avoid toxic, inflammatory, or outflow-obstructing fragments.
Diffusion behavior	Diffusion-dominated, close to native/porcine vitreous for oxygen, metabolites, and drugs	Avoids excessive convection while allowing nutrition, waste removal, and predictable pharmacokinetics.

**Table 2:** Comparative properties and limitations of current clinical vitreous substitutes. The table compares gases, standard silicone oil, high-viscosity silicone oil, heavy silicone oil, semi-fluorinated alkane-containing formulations, PFCLs, balanced salt solution, and experimental hydrogels in terms of optical behavior, density, residence time, handling, biological effects, and oxygen/lens considerations.

Substitute	Examples/Composition	Density/Buoyancy	Viscosity/Refractive Behavior	Typical Residence	Main Advantage	Main Limitations	Oxygen/Lens Considerations
Air/expansile gases	Air, SF_6_, C_3_F_8_	Very low density; buoyant	Gas phase; refractive mismatch during filling	Days to weeks-months	Short-term superior tamponade	Positioning, expansion, no matrix function	No durable oxygen buffering after resorption
Standard silicone oil	Mostly 1000-cSt PDMS	Lighter than water	High RI (~1.404); low gel-like elasticity	Months; usually removed	Durable tamponade and visibility	Emulsification, glaucoma, keratopathy, retinal changes	Alters oxygen distribution but does not restore gel barrier
High-viscosity silicone oil	Mostly 5000-cSt PDMS	Lighter than water	Higher viscosity; similar RI	Months; usually removed	May reduce emulsification tendency	Harder injection/removal; still not physiological	Same non-physiological diffusion profile
Heavy silicone oil	PDMS plus fluorinated components	Heavier than water	Density adjusted; RI variable	Short-to-medium term	Improves inferior tamponade	Inflammation, emulsification, difficult removal	May increase inferior interface stress
Semi-fluorinated alkane systems	F6H8/F6H5-related mixtures/additives	Density tuning possible	Useful solubility/density modifiers	Selected temporary uses	Improves handling or heavy-oil behavior	Droplet toxicity, incomplete mixing, degradation concerns	Lens effects uncertain; no oxygen-buffering function
PFCLs	Perfluorodecalin, perfluoro-n-octane	Heavy	Low viscosity, high density	Intraoperative/short term	Excellent retinal flattening	Retained droplets, macrophage response, retinal toxicity	Not a long-term oxygen regulator
Balanced salt solution	Physiological aqueous irrigant	Neutral	Aqueous; no viscoelasticity	Minutes to hours	Maintains volume during surgery	No support or biochemical protection	Rapid equilibration; no lens protection
Experimental hydrogels	HA, PEG, hybrid, bioactive gels	Usually neutral	RI close to vitreous; tunable G′	Weeks to long-term target	Biomimetic mechanics and diffusion	Swelling, degradation, regulation, validation	Potential to recreate diffusion barrier, unproven clinically

**Table 3:** Hydrogel and polymer-based vitreous substitutes under preclinical or early clinical investigation. The table is organized by material class, crosslinking or gelation strategy, strengths, limitations, and translational stage.

Material Class	Representative Systems	Crosslinking/Gelation	Main Strengths	Key Limitations	Evidence Stage
Natural polymers	HA hydrogels, modified HA, alginate-HA, chitosan-HA	Chemical, dynamic covalent, enzymatic, UV or self-crosslinking	Native-matrix similarity, clarity, injectability, cytocompatibility	Enzymatic degradation, swelling, variable durability	In vitro, rabbit, phthisis-bulbi experience
Self-assembling peptides	Peptide nanofiber gels	Supramolecular self-assembly	ECM-like nanofibers, high transparency	Cost, scale-up, long-term stability	Rabbit studies
Gelatin/GelMA systems	GelMA and modified gelatin matrices	Photopolymerization or dynamic bonds	Cell-compatible network, tunable mechanics	Light exposure, crosslinker safety, degradation	Preclinical retinal surgery models
Synthetic polymers	PEG, PNIPAAm, poloxamers	Photo/chemical crosslinking or thermogelling	Precise chemistry, tunable mechanics, drug release	Non-native degradation products, regulatory burden	In vitro, rabbit, primate for selected gels
Hybrid/composite systems	HA-PEG IPNs, HA-agar, GelMA-nanofiber composites	Dual networks, non-covalent blends, nanoreinforcement	Improved mechanics, slower degradation, diffusion tuning	Higher formulation complexity and batch control	Preclinical and in vitro diffusion models
Bioactive hydrogels	Vitamin C, EGCG, steroid, anti-VEGF, NRF2-active polymers	Drug-loaded, antioxidant, or pathway-modulating networks	Disease-specific ROS buffering, anti-inflammatory or anti-fibrotic activity	Dose control, off-target effects, indication specificity	In vitro and animal models

**Table 4:** Functional gaps and future requirements for vitreous substitutes. The table contrasts the main limitations of current clinical substitutes, including gases, silicone oils, and perfluorocarbon liquids, with the functional objectives expected from next-generation vitreous replacements across mechanical, metabolic, optical, and biocompatibility domains.

Functional Domain	Main Gap in Current Clinical Substitutes	Possible Consequence After Vitrectomy	Future Requirement for Next-Generation Substitutes
Mechanical and rheological support	Gases, PFCLs, and silicone oils provide tamponade mainly through buoyancy, density, or surface tension, but do not reproduce native vitreous viscoelastic damping.	Incomplete force damping, altered intraocular fluid movement, and possible abnormal shear at the vitreoretinal interface.	Soft, transparent, injectable gels with storage modulus, viscosity, and deformation recovery close to the physiological vitreous range.
Optical and refractive performance	Most current agents are optically useful for surgery but may cause refractive shift, optical interfaces, droplets, opacification, or phase separation over time.	Reduced visual quality, glare, image distortion, or need for substitute removal.	Long-term visible-light transmittance, refractive index close to vitreous, resistance to opacification, and absence of particle formation.
Oxygen-gradient and metabolic buffering	Removal of the native gel increases oxygen mixing; gases, BSS, and oils do not recreate the controlled diffusion behavior of native vitreous.	Higher oxygen delivery to the lens may contribute to nuclear cataract progression; altered retinal metabolic exchange may also occur.	Diffusion-regulating matrices able to limit excessive convection while permitting physiological oxygen, nutrient, metabolite, and drug transport.
Biochemical and disease-specific bioactivity	Conventional tamponades are largely inert and do not modulate oxidative stress, inflammation, angiogenic drive, or fibrotic signaling.	Persistent oxidative or inflammatory stress may favor retinal injury, diabetic complications, or proliferative vitreoretinopathy in susceptible eyes.	Optional, indication-specific antioxidant, anti-inflammatory, anti-VEGF, or anti-fibrotic functions, rather than universal requirements for all cases.
Biocompatibility and long-term safety	Emulsification, retained droplets, inflammatory reaction, glaucoma, keratopathy, retinal toxicity, or difficult removal may occur with established materials.	Chronic complications may limit residence time and require secondary surgery.	Low cytotoxicity, minimal protein fouling and cell adhesion, stable degradation products, preserved retinal structure and function, and controlled IOP response.
Manufacturing and clinical translation	Current experimental hydrogels often lack standardized testing, scalable production, and validated long-term performance across models.	Promising in vitro behavior may not predict long-term human ocular safety or efficacy.	Reproducible GMP-compatible synthesis, standardized optical/rheological/diffusion testing, appropriate animal models, and cautious early-phase clinical trials.

## Data Availability

No new data were created or analyzed in this study. Data sharing is not applicable to this article.
